# Supplementary figures and images for: Therapeutic concentrations of calcineurin inhibitors do not deregulate glutathione redox balance in human renal proximal tubule cells
Source: PLoS One. 2021 Apr 30;16(4):e0250996. doi: 10.1371/journal.pone.0250996 (PMC8087105; doi:10.1371/journal.pone.0250996)

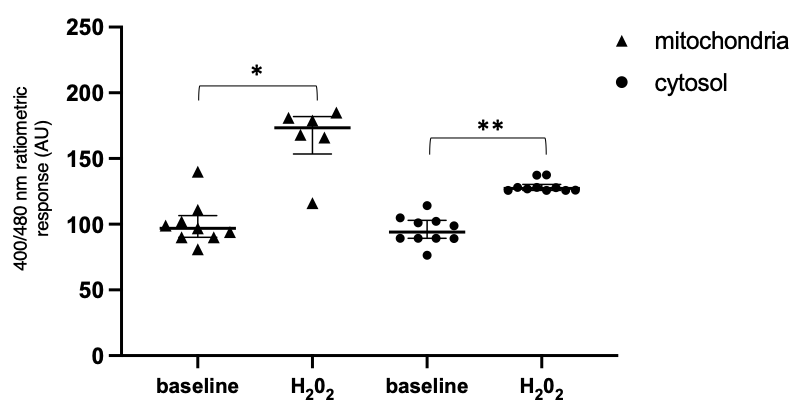

Supplement: S1 Fig — CiPTC expressing mitochondrial or cytosolic roGFP2 were exposed or not to 1 mM H2O2 for 15 min. The large and small horizontal lines show the median and standard deviations, respectively. Every individual symbol represents the average ratio of 10 measurements within one cell (at least 6 randomly chosen cells were analyzed per experiment). The Wilcoxon test was used to calculate p-values, and statistical differences are marked (*, p < 0.05; **, p < 0.01). (TIF) [file pone.0250996.s001.tif]

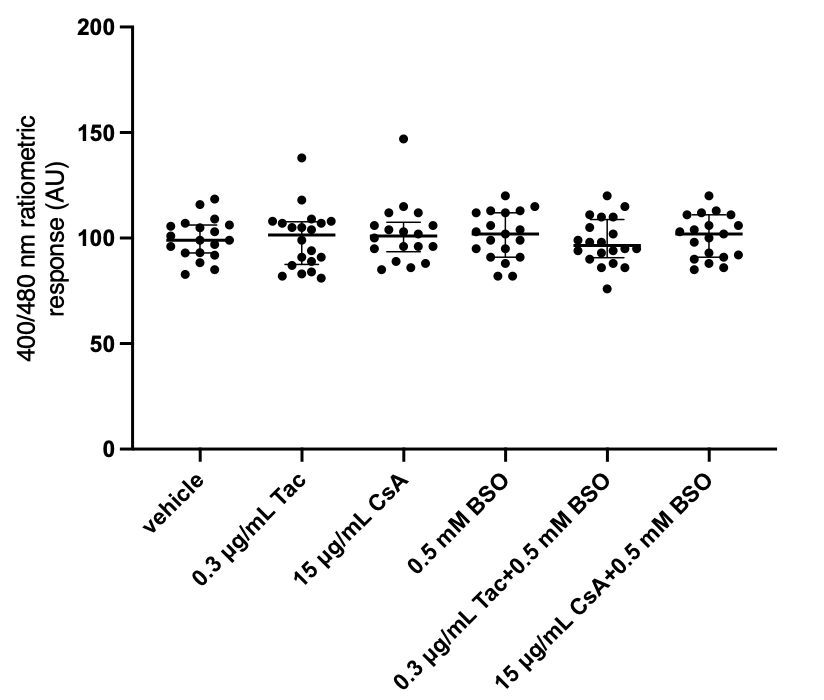

Supplement: S2 Fig — CiPTC expressing cytosolic roGFP2 were exposed for 48 h to vehicle (0.1% v/v DMSO), 15 μg/mL of cyclosporin A (CsA), 0.3 μg/mL tacrolimus (Tac), 0.5 mM L-buthionine-sulfoximine (BSO), or a combination of CsA or Tac with BSO. The large and small horizontal lines show the median and standard deviations, respectively. Every individual symbol represents the average) ratio of 10 measurements within one cell (at least 9 randomly chosen cells were analyzed per experiment; number of independent experiments: 2). Data were statistically analyzed using one-way ANOVA, but no significant differences were detected. (TIF) [file pone.0250996.s002.tif]

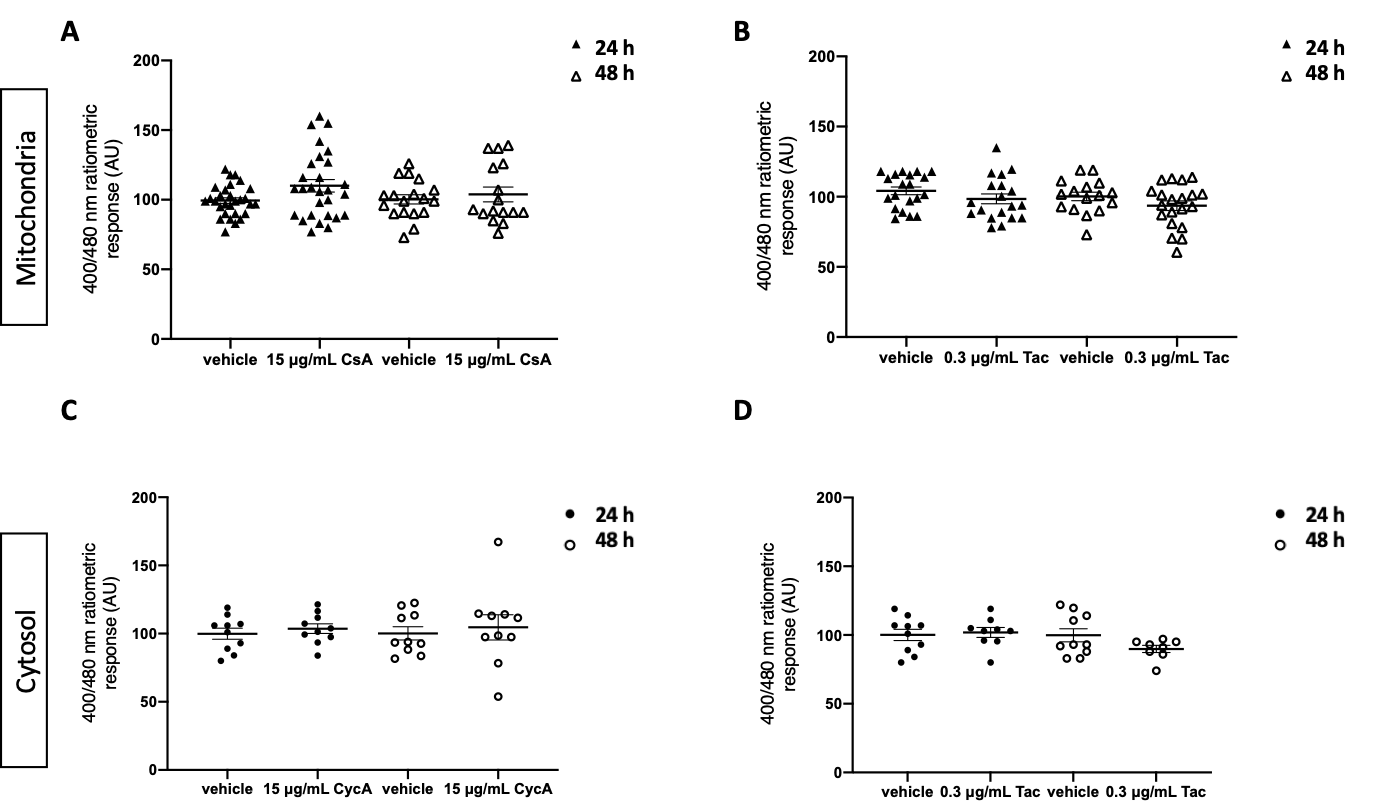

Supplement: S3 Fig — CiPTC expressing mitochondrial or cytosolic roGFP2-Orp1 were exposed to (A, C) vehicle (0.1% v/v DMSO) or 15 μg/mL of cyclosporin A (CsA) and (B, D) vehicle or 0.3 μg/mL tacrolimus (Tac). The upper and lower panels summarize the results of 3 and 1 experiment, respectively. The large and small horizontal lines show the median and standard deviations (as error bars), respectively. Every individual symbol depicts the average ratio of 10 measurements within one cell (at least 5 randomly chosen cells were analyzed per experiment). One-way ANOVA test was used to calculate p-values: (A) 0.1; (B) 0.1, (C) 0.9; and (D) 0.1. (TIF) [file pone.0250996.s003.tif]

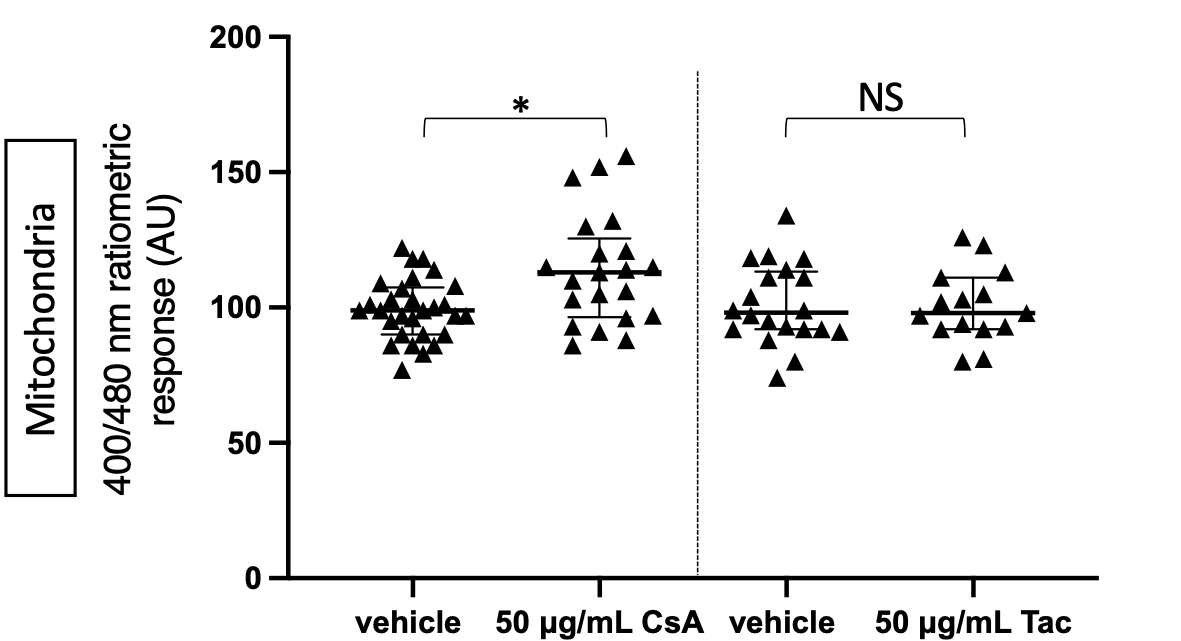

Supplement: S4 Fig — ciPTC expressing mitochondrial roGFP2-Orp1 were exposed for 24 h to vehicle (for CsA: 0.1% v/v DMSO; for Tac: 0.5% v/v DMSO), 50 μg/mL CsA, or 50 μg/ml Tac. The results are derived from 3 independent experiments. The large and small horizontal lines show the median and standard deviations (as error bars), respectively. Every individual symbol depicts the average ratio of 10 measurements within one cell (at least 5 randomly chosen cells were analyzed per experiment). The paired t-test was used to calculate p-values: *, 0.01; non-significant (NS), 0.6. (TIF) [file pone.0250996.s004.tif]
