# Supplementary material for: Therapeutic concentrations of calcineurin inhibitors do not deregulate glutathione redox balance in human renal proximal tubule cells
Source: PLoS One. 2021 Apr 30;16(4):e0250996. doi: 10.1371/journal.pone.0250996 (PMC8087105; doi:10.1371/journal.pone.0250996)
Supplement: S1 Table — For experimental details, see legend to Fig 1. The percentages represent the mean 400/480 nm ratiometric response ratios compared to the averages of the corresponding non-treated control cells, which were set to 100%. Given that peroxisomes have the capacity to resist oxidative insults generated outside the organelle [1], cells expressing peroxisomal roGFP2 (PO) were not challenged with external H2O2. The Kruskal-Wallis (KW) or One-way ANOVA (OWA) test were used to calculate p-values. C, cytosolic roGFP2; ciPTC, conditionally immortalized proximal tubule cell; CsA, cyclosporin A; MT, mitochondrial roGFP2; Tac, tacrolimus. (PDF) [file pone.0250996.s005.pdf]

Treatment		CsA (15 μg/mL)		Tac (0.3 μg/mL)		CsA (50 μg/mL)	Tac (50 μg/mL)	H_2O_2 (1 mM)
Time point (h)		**24**	**48**	**24**	**48**	**24**	**24**	**0.25**
**MT**	% of control	105	101	101	97	112	108	166
	p-value	0.8 (KW)		0.4 (KW)		0.02 (OWA)	0.2 (OWA)	0.03 (OWA)
**PO**	% of control	107	97	99	95			
	p-value	0.2 (KW)		0.6 (KW)				
**C**	% of control	101	98	93	97			129
	p-value	0.8 (KW)		0.2 (KW)				0.002 (OWA)
