# Supplementary material for: Therapeutic concentrations of calcineurin inhibitors do not deregulate glutathione redox balance in human renal proximal tubule cells
Source: PLoS One. 2021 Apr 30;16(4):e0250996. doi: 10.1371/journal.pone.0250996 (PMC8087105; doi:10.1371/journal.pone.0250996)
Supplement: S2 Table — (PDF) [file pone.0250996.s006.pdf]

S2 Table. Quantitative analyses of mitochondrial morphology in ciPTC under CNI.

Treatment	Total cells counted n=1, 2, 3	Cells with elongated mitochondria n=1, 2, 3	% of cells with elongated mitochondria n=1, 2, 3	Cells with fragmented mitochondria n=1, 2, 3	% of cells with fragmented mitochondria n=1, 2, 3	p-value
0.1% DMSO	6, 9, 12	6, 8, 8	100, 88.9, 66.7	0, 1, 4	0, 11.1, 33.3	
15 μg/mL CsA	9, 16, 20	6, 14, 16	66.7, 87.5, 80	3, 2, 4	33.3, 12.5, 20.0	vs. 0.1% DMSO: 0.288
50 μg/mL CsA	4, 11, 8	1, 8, 3	25, 72.8, 37.5	3, 3, 5	75.0, 27.2, 62.5	vs. 0.1% DMSO: 0.043
0.3 μg/mL Tac	8, 12, 21	5, 11, 16	62.5, 91.6, 76.2	3, 1, 5	37.5, 8.3, 23.8	vs. 0.1% DMSO: 0.276
0.5% DMSO	10, 14, 15	6, 8, 8	60, 57.1, 53.3	4, 6, 7	40.0, 42.8, 46.7	vs. 0.1% DMSO: 0.048
50 μg/mL Tac	5, 12, 12	0, 4, 2	0, 33.3, 16.7	5, 8, 10	100.0, 66.7, 83.3	vs. 0.5% DMSO: 0.024

All percentages are volume-based. ciPTC, conditionally immortalized proximal tubule cell; CsA, cyclosporine A; Tac, tacrolimus.
