# Supplementary material for: Therapeutic concentrations of calcineurin inhibitors do not deregulate glutathione redox balance in human renal proximal tubule cells
Source: PLoS One. 2021 Apr 30;16(4):e0250996. doi: 10.1371/journal.pone.0250996 (PMC8087105; doi:10.1371/journal.pone.0250996)
Supplement: S3 Table — (PDF) [file pone.0250996.s007.pdf]

S3 Table. Details of the articles studying ROS production in response to CNI in PTC with different origins.

Cell line	Method	Concentration of CNI	Time window	Vehicle	Results	Reference	References for CYP3A5 expression	References for P-gp expression
HK-2	Amplex Red assay	8 µg/mL CsA<br>8.3 µg/mL Tac	24 h	ethanol	higher H_2O_2 in CsA vs. Tac	[2]	[3]	[4],[5]
HK-2	DCFH-DA probe (staining)	8 µg/mL CsA	24 h	no info	higher staining in CsA than control	[6]		
HK-2	MitoSOX (flow cytometry)	50 µg/mL Tac	12 h	no info	higher staining in Tac-treated cells	[7]		
HK-2	MitoSOX (staining)	60 µg/mL Tac	12 h	no info	higher staining in Tac-treated cells	[8]		
LLC-PK_1	DCFH-DA probe (flow cytometry)	50 µg/mL Tac<br>66.6 µg/mL Tac	40 min	DMSO	higher H_2O_2 in Tac-treated cells	[9]	No references in PubMed	[10]
Primary mouse PTC	MitoSOX (staining)	20 µg/mL CsA	24 h	ethanol	no ROS detected	[11]	No references in PubMed	[12]

Specific PTC characteristics of HK-2 are not confirmed [13, 14]. Amplex Red, peroxide detection assay; CsA, cyclosporine A; HK-2, immortalized human proximal tubule cells; LLC-PK1, immortalized porcine proximal tubule cells; MitoSOX, mitochondrial superoxide detection assay; P-gp, P-glycoprotein; ROS, reactive oxygen species; Tac, tacrolimus.
