# Supplementary material for: Therapeutic concentrations of calcineurin inhibitors do not deregulate glutathione redox balance in human renal proximal tubule cells
Source: PLoS One. 2021 Apr 30;16(4):e0250996. doi: 10.1371/journal.pone.0250996 (PMC8087105; doi:10.1371/journal.pone.0250996)
Supplement: S4 Table — For experimental details, see legend to Fig 1. The percentages represent the mean 400/480 nm ratiometric response ratios compared to the averages of the corresponding non-treated control cells, which were set to 100%. Given that, under basal conditions, peroxisomal roGFP2-Orp1 is almost fully oxidized in ciPTC, this sensor was not included in this analysis. One-way ANOVA (OWA) test were used to calculate p-values for more than 2 groups. Paired t-test (PPT) and Wilcoxon test (WT) were used to calculate the difference between 2 groups. C, cytosolic roGFP2; ciPTC, conditionally immortalized proximal tubule cell; CsA, cyclosporin A; MT, mitochondrial roGFP2; Tac, tacrolimus. (PDF) [file pone.0250996.s008.pdf]

S4 Table. Mean normalized values of the mitochondrial and cytosolic roGFP2-Orp1-based H_2O_2 sensors in ciPTC exposed to calcineurin inhibitors or hydrogen peroxide. For experimental details, see legend to Fig 1. The percentages represent the mean 400/480 nm ratiometric response ratios compared to the averages of the corresponding non-treated control cells, which were set to 100%. Given that, under basal conditions, peroxisomal roGFP2-Orp1 is almost fully oxidized in ciPTC, this sensor was not included in this analysis. One-way ANOVA (OWA) test were used to calculate p-values for more than 2 groups. Paired t-test (PPT) and Wilcoxon test (WT) were used to calculate the difference between 2 groups. C, cytosolic roGFP2; ciPTC, conditionally immortalized proximal tubule cell; CsA, cyclosporin A; MT, mitochondrial roGFP2; Tac, tacrolimus.

**Treatment**		**CsA (15 μg/mL)**		**Tac (0.3 μg/mL)**		**CsA (50 μg/mL)**	**Tac (50 μg/mL)**	**H_2O_2 (1 mM)**
**Time point (h)**		**24**	**48**	**24**	**48**	**24**	**24**	**0.25**
**MT**	% of control	110	104	98	94	114	101	
	p-value	0.1 (OWA)		0.1 (OWA)		0.01 (PPT)	0.6 (PPT)	
**C**	% of control	104	105	102	90			178
	p-value	0.9 (OWA)		0.1 (OWA)				0.007 (WT)
