# Supplementary material for: Therapeutic concentrations of calcineurin inhibitors do not deregulate glutathione redox balance in human renal proximal tubule cells
Source: PLoS One. 2021 Apr 30;16(4):e0250996. doi: 10.1371/journal.pone.0250996 (PMC8087105; doi:10.1371/journal.pone.0250996)
Supplement: S1 References — (PDF) [file pone.0250996.s009.pdf]

References

1. Ivashchenko O, Van Veldhoven PP, Brees C, Ho YS, Terlecky SR, Fransen M. Intraperoxisomalredox balance in mammalian cells: oxidative stress and interorganellar cross-talk. Mol Biol Cell. 2011;22(9):1440-51.
2. Koppelstaetter C, Kern G, Leierer G, Mair SM, Mayer G, Leierer J. Effect of cyclosporine, tacrolimus and sirolimus on cellular senescence in renal epithelial cells. Toxicol In Vitro. 2018;48:86-92.
3. Dai Y, Hebert MF, Isoherranen N, Davis CL, Marsh C, Shen DD, et al. Effect of CYP3A5 polymorphism on tacrolimus metabolic clearance in vitro. Drug Metab Dispos. 2006;34(5):836-47.
4. Romiti N, Tramonti G, Chieli E. Influence of different chemicals on MDR-1 P-glycoprotein expression and activity in the HK-2 proximal tubular cell line. Toxicol Appl Pharmacol. 2002;183(2):83-91.
5. Zager RA. P glycoprotein-mediated cholesterol cycling determines proximal tubular cell viability. Kidney Int. 2001;60(3):944-56.
6. Lai Q, Luo Z, Wu C, Lai S, Wei H, Li T, et al. Attenuation of cyclosporine A induced nephrotoxicity by schisandrin B through suppression of oxidative stress, apoptosis and autophagy. Int Immunopharmacol. 2017;52:15-23.
7. Yu JH, Lim SW, Luo K, Cui S, Quan Y, Shin YJ, et al. Coenzyme Q(10) alleviates tacrolimus-induced mitochondrial dysfunction in kidney. Faseb j. 2019;33(11):12288-98.
8. Lim SW, Jin L, Luo K, Jin J, Shin YJ, Hong SY, et al. Klotho enhances FoxO3-mediated manganese superoxide dismutase expression by negatively regulating PI3K/AKT pathway during tacrolimus-induced oxidative stress. Cell Death Dis. 2017;8(8):e2972.

9. Zhou X, Yang G, Davis CA, Doi SQ, Hirszel P, Wingo CS, et al. Hydrogen peroxide mediates FK506-induced cytotoxicity in renal cells. Kidney Int. 2004;65(1):139-47.
10. Magnarin M, Morelli M, Rosati A, Bartoli F, Candussio L, Giraldi T, et al. Induction of proteins involved in multidrug resistance (P-glycoprotein, MRP1, MRP2, LRP) and of CYP 3A4 by rifampicin in LLC-PK1 cells. Eur J Pharmacol. 2004;483(1):19-28.
11. Kimura T, Takahashi A, Takabatake Y, Namba T, Yamamoto T, Kaimori JY, et al. Autophagy protects kidney proximal tubule epithelial cells from mitochondrial metabolic stress. Autophagy. 2013;9(11):1876-86.
12. Tsuruoka S, Sugimoto KI, Fujimura A, Imai M, Asano Y, Muto S. P-glycoprotein-mediated drug secretion in mouse proximal tubule perfused in vitro. J Am Soc Nephrol. 2001;12(1):177-81.
13. Bajaj P, Chowdhury SK, Yucha R, Kelly EJ, Xiao G. Emerging kidney models to investigate metabolism, transport, and toxicity of drugs and xenobiotics. Drug Metab Dispos. 2018;46(11):1692-702.
14. Jenkinson SE, Chung GW, van Loon E, Bakar NS, Dalzell AM, Brown CD. The limitations of renal epithelial cell line HK-2 as a model of drug transporter expression and function in the proximal tubule. Pflugers Arch. 2012;464(6):601-11.
